# Social disparities in pain management provision in stage IV lung cancer: A national registry analysis

**DOI:** 10.1097/MD.0000000000032888

**Published:** 2023-02-22

**Authors:** Marita Yaghi, Najla Beydoun, Kelsey Mowery, Sandra Abadir, Maroun Bou Zerdan, Iktej Singh Jabbal, Carlos Rivera, Hong Liang, Evan Alley, Diana Saravia, Rafael Arteta-Bulos

**Affiliations:** a Department of Hematology/Oncology, Maroone Cancer Center, Cleveland Clinic Florida, Weston, FL; b Department of Anesthesiology and Perioperative Medicine, Tufts Medical Center, Boston, MA; c Ross University School of Medicine, Bridgetown, BRB; d Department of Internal Medicine, SUNY Upstate Medical University, Syracuse, NY.

**Keywords:** carcinoma, healthcare disparities, non-small-cell lung, pain management, quality of life, small-cell lung carcinoma

## Abstract

A strong association exists between pain and lung cancer (LC). Focusing on the disparities in pain referral in LC patients, we are aiming to characterize the prevalence and patterns of referrals to pain management (PM) in Stage IV non-small-cell LC (NSLC) and small-cell LC (SCLC). We sampled the National Cancer Database for de novo stage IV LC (2004–2016). We analyzed trends of pain referral using the Cochran–Armitage test. Chi-squared statistics were used to identify the sociodemographic and clinico-pathologic determinants of referral to PM, and significant variables (*P* < .05) were included in one multivariable regression model predicting the likelihood of pain referral. A total N = 17,620 (3.1%) of NSLC and N = 4305 (2.9%) SCLC patients were referred to PM. A significant increase in referrals was observed between 2004 and 2016 (NSLC: 1.7%–4.1%, *P* < .001; SCLC: 1.6%–4.2%, *P* < .001). Patient and disease factors played a significant role in likelihood of referral in both groups. Demographic factors such as gender, age, and facility type played a role in the likelihood of pain referrals, highlighting the gap and need for multidisciplinary PM in patients with LC. Despite an increase in the proportion of referrals to PM issued for terminal stage LC, the overall proportion remains low. To ensure better of quality of life for patients, oncologists need to be made aware of existent disparities and implicit biases.

## 1. Introduction

Pain in cancer patients is often devastating. It severely affects patient comfort, motivation, interaction with friends and family, and overall quality of life. Pain is reported in 59% of patients undergoing cancer treatment, and in 64% of patients with advanced disease.^[1]^

Lung cancer (LC) is the second most common non-skin cancer in both men and women in the US.^[2,3]^ Most LCs are metastatic at diagnosis,^[4]^ hence, the importance of palliative and comfort care is undeniable. Indeed, patients with LC often experience high levels of pain.^[5,6]^ Primary LC may cause localized chest pain, as these solitary tumors may involve the ribs, parietal pleura, thoracic spinal cord, or brachial plexus. Pain that is caused by LC may either be a result of mediastinal invasion due to nerve entrapment, compression or invasion of the esophagus, or vascular obstruction.^[7]^ Superior vena cava syndrome and Pancoast syndrome, common complications of locally advanced LC, may manifest with pain localized to the shoulder and medial scapular border, as well as radicular pain along the ulnar distribution.^[7]^ Furthermore, LC has the propensity to metastasize to the liver, bones and brain, causing distant pain through destruction or compression of the affected distant sites. Additionally, patients with LC who undergo either radiotherapy and/or chemotherapy, may develop pain as an adverse effect from treatment.^[8–11]^ Radiation therapy in these patients can cause radiation-induced esophagitis, odynophagia, esophageal strictures, radiation pneumonitis, radiation-induced pericarditis or rarely, osteoradionecrosis.^[12]^ Conversely, chemotherapy-induced pain may be a result of interstitial pneumonitis and peripheral neuropathy.^[13–15]^

Disparities in pain management (PM) have been well documented. Inequalities in pain reporting and adequate treatment for acute and/or chronic pain have been shown to affect minority individuals.^[16]^ Black Americans, Hispanics or patients from underserved communities that are diagnosed with LC, face worse outcomes when compared to White Americans.^[17]^ Prior investigations have shown that these patients with LC are less likely to be diagnosed early, less likely to receive surgical treatment, and/or are more likely to not receive any treatment.^[2,17–20]^ A variety of factors that may be linked to the increased burden of LC in minority populations include increased risk of smoking-induced LC, poor access to health care services, and lower success rates in smoking cessation attempts.^[21–27]^

Currently, no data exists regarding the disparities in PM provision in patients with LC. Therefore, our goal was to determine the prevalence, patterns and determinants of disparities in PM referrals in patients with stage IV small cell LC (SCLC) and non-SCLC (NSCLC), on a national level.

## 2. Methods

### 2.1. Patient data

We retrospectively analyzed a sample of patients from the National Cancer Database (NCDB). In this observational study, patients with de novo stage IV SCLC and NSCLC diagnosed between 2004 and 2016 were included. Access to the NCDB was achieved based on a Participant User File award granted to the principal investigator (R.A.B). The NCDB is a large de-identified national database in the US, jointly supported by the American College of Surgeons and the Commission on Cancer. It encompasses an estimated 70% of all cancer diagnoses from >1500 institutions nationwide.^[28]^

### 2.2. Statistical analysis

Data analysis was done using version 27.0 of the Statistical Package for the Social Sciences (SPSS) software (IBM, Version 27.0. Armonk, NY: IBM Corp). In both groups of patients – those diagnosed with SCLC and those diagnosed with NSCLC – we identified 2 cohorts by the status of referral to pain specialists: patients who received referral to pain specialists regardless of other palliative treatment (chemotherapy, radiotherapy, immunotherapy, or surgery), and patients who did not receive referral to pain specialists regardless of other palliative treatment.

Using the Cochran–Armitage test, we determined the trends of PM referral amongst both patient groups, looking at the proportion of patients referred every year. Bivariate analysis with chi-squared statistics was then performed to compare patient sociodemographic (including age, sex, race, etc) and clinicopathologic characteristics (such as Charlson/Deyo comorbidity scoring, treatment, metastatic sites, etc) between 2 patient groups, based on PM referral status. Finally, a multivariate regression model examined the impact of major demographic predictors on the likelihood of receiving PM referral.

## 3. Results

### 3.1. Trends of referral for PM

A total of N = 565,884 patients with stage IV NSCLC and 148,584 patients with stage IV SCLC were included in this analysis.

**Figure 1.**
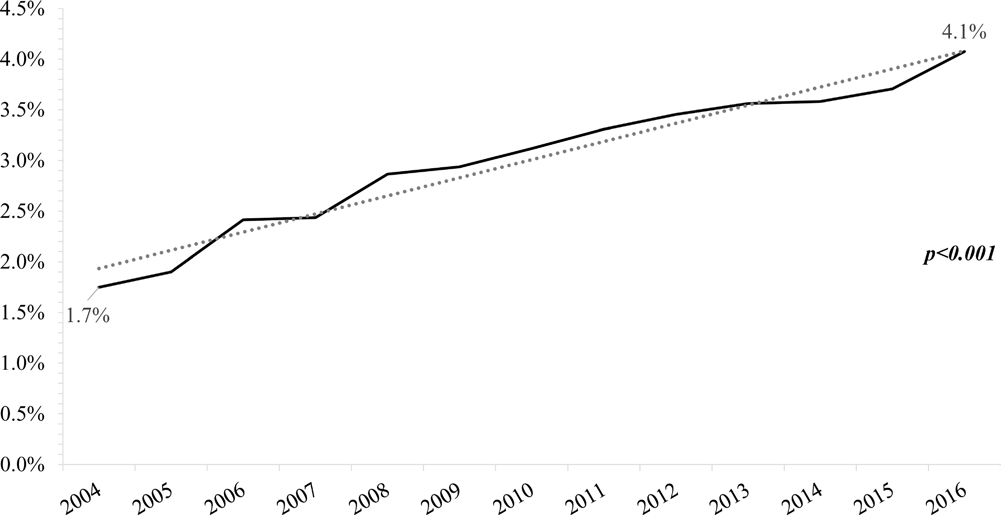
Proportion of stage IV non-small cell lung cancer (NSCLC) patients referred to pain management.

A total of 3.1% of patients with de novo stage IV NSCLC were referred to pain specialists (Table 1). The proportion of patients referred per year showed a significant increase over time: 1.7% of patients diagnosed in 2004 were referred for PM versus 4.1% of patients in 2016 (*P* < .001) (Fig. 1).

**Table 1:** Sociodemographic and clinicopathologic determinants of referral for pain management in non-small cell lung cancer.

Variable	Referred to pain management N = 565,884		Chi-squared *P* value	Multiple logistic regression	
	No, N = 548,264 (96.9%)	Yes, N = 17,620 (3.1%)		OR (95% CI)	*P* value
Sex					
Male (ref)	301,636 (96.8.0%)	9872 (3.2%)	.008	1	–
Female	246,628 (97.0%)	7748 (3.0%)		0.98 (0.95–1.01)	.128
Age					<.001
<50 (ref)	33,335 (96.6%)	1180 (3.4%)	<.001	1	–
50–70	286,787 (96.8%)	9423 (3.2%)		0.90 (0.84–0.96)	.002
>70	228,142 (97.0%)	7017 (3.0%)		0.77 (0.71–0.83)	<.001
Race					<.001
White	442,950 (96.8%)	14,776 (3.2%)	<.001	1	–
Black	66,500 (97.5%)	1709 (2.5%)		0.73 (0.69–0.77)	<.001
Asian	1398 (96.6%)	49 (3.4%)		0.91 (0.67–1.23)	.534
Native American	14,390 (96.6%)	504 (3.4%)		1.10 (1.00–1.21)	.053
Pacific islanders	360 (94.7%)	20 (5.3%)		1.76 (1.12–2.77)	.015
Other	15,689 (97.7%)	363 (2.3%)		0.46 (0.17–1.28)	.139
Hispanic					
Non-Hispanic	498,800 (96.8%)	16,469 (3.2%)	<.001	1	–
Hispanic	16,853 (97.6%)	407 (2.4%)		1.43 (0.52–3.95)	.490
Insurance					<.001
Not insured	23,311 (96.4%)	872 (3.6%)	<.001	1	–
Private	156,032 (97.2%)	4444 (2.8%)		0.81 (0.75–0.88)	<.001
Medicaid*	41,272 (96.0%)	1709 (4.0%)		1.11 (1.01–1.21)	.023
Medicare	317,765 (96.8%)	10,369 (3.2%)		0.94 (0.87–1.02)	.135
Charlson/Deyo score					<.001
0	340,715 (97.2%)	9641 (2.8%)	<.001	1	–
1	137,083 (96.4%)	5108 (3.6%)		1.32 (1.27–1.36)	<.001
2	48,244 (96.2%)	1890 (3.8%)		1.38 (1.32–1.46)	<.001
3	22,222 (95.8%)	981 (4.2%)		1.56 (1.46–1.67)	<.001
Facility setting					<.001
Metro	441,130 (97.1%)	13,331 (2.9%)	<.001	1	–
Urban	82,166 (96.0%)	3444 (4.0%)		1.37 (1.31–1.42)	<.001
Rural	11,556 (95.6%)	524 (4.4%)		1.45 (1.31–1.59)	<.001
Facility type					<.001
CCP	62,232 (96.9%)	1991 (3.1%)	<.001	1	–
CoCCP	241,457 (96.9%)	7731 (3.1%)		1.06 (1.01–1.12)	.022
ACP	164,347 (96.6%)	5801 (3.4%)		1.24 (1.17–1.31)	<.001
INCP	76,203 (97.5%)	1971 (2.5%)		0.88 (0.82–0.94)	<.001
Surgery at primary site					
No	530,152 (96.8%)	17,364 (3.2%)	<.001	1	–
Yes	17,031 (98.6%)	246 (1.4%)		0.45 (0.40–0.52)	<.001
Surgery at secondary site					
No	513,475 (96.9%)	16,458 (3.1%)	.132	–	–
Yes	34,151 (96.8%)	1147 (3.2%)		–	–
Radiation					
No	286,187 (97.3%)	8057 (2.7%)	<.001	1	–
Yes	262,077 (96.5%)	9563 (3.5%)		1.32 (1.28–1.37)	<.001
Chemotherapy					
No	238,614 (96.1%)	9739 (3.9%)	<.001	1	–
Yes	293,556 (97.5%)	7610 (2.5%)		0.60 (0.58–0.62)	<.001
Immunotherapy					
No	528,563 (96.9%)	17,015 (3.1%)	.366	–	–
Yes	17,636 (96.8%)	590 (3.2%)		–	–
Metastasis site					<.001
Bone only	10,836 (94.6%)	614 (5.4%)	<.001	1	–
Brain only	8040 (97.3%)	224 (2.7%)		0.49 (0.42–0.58)	<.001
Liver only	2899 (96.8%)	97 (3.2%)		0.59 (0.48–0.73)	<.001
Number of metastatic sites					<.001
1 site only	21,775 (95.9%)	935 (4.1%)	<.001	1	–
2 sites	7426 (93.8%)	493 (6.2%)		1.55 (1.38–1.73)	<.001
3 sites	1545 (93.7%)	103 (6.3%)		1.55 (1.26–1.92)	<.001

ACP = Academic/Research Cancer Program, CCP = Community Cancer Program, CoCCP = Comprehensive Community Cancer Program, CI = confidence interval, INCP = Integrated Network Cancer Program, OR = odds ratio.

* Medicaid and other governmental programs.

**Figure 2.**
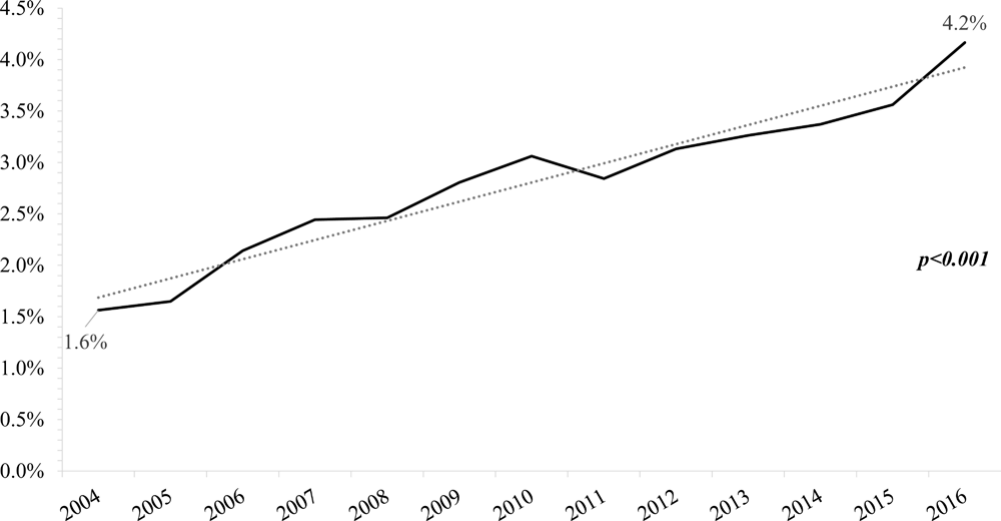
Proportion of stage IV small cell lung cancer (SCLC) patients referred to pain management.

In patients with stage IV SCLC, 2.9% received referrals overall (Table 2). A significant increase in PM referrals was also noted, with a proportion of 1.6% of referrals for patients diagnosed in 2004 versus 4.2% in 2016 (*P* < .001) (Fig. 2).

**Table 2:** Sociodemographic and clinicopathologic determinants of referral for pain management in small cell lung cancer.

Variable	Referred to pain management N = 148,584		Chi-squared *P* value	Multiple logistic regression	
	No, N = 144,279 (97.1%)	Yes, N = 4305 (2.9%)		OR (95% CI)	*P* value
Sex					
Male (ref)	74,108 (97.1%)	2206 (2.9%)	.875	–	–
Female	70,171 (97.1%)	2099 (2.9%)		–	–
Age					
<50 (ref)	6467 (96.9%)	208 (3.1%)	.478	–	–
50–70	83,018 (97.1%)	2451 (2.9%)		–	–
>70	54,794 (97.1%)	1646 (2.9%)		–	–
Race					.944
White	130,092 (97.1%)	3941 (2.9%)	<.001	1	–
Black	10,799 (97.7%)	257 (2.3%)		0.80 (0.53–1.21)	.287
Asian	1369 (96.9%)	44 (3.1%)		0.88 (0.32–2.41)	.799
Native American	397 (96.1%)	16 (3.9%)		NA	.998
Pacific islanders	134 (90.5%)	14 (9.5%)		NA	.999
Other	1488 (97.8%)	33 (2.2%)		1.07 (0.39–2.95)	.900
Hispanic					
Non-Hispanic	131,853 (97.0%)	4061 (3.0%)	.060	–	–
Hispanic	3290 (97.6%)	82 (2.4%)		–	–
Insurance					.086
Not insured	5905 (96.8%)	193 (3.2%)	<.001	1	–
Private	38,637 (97.6%)	962 (2.4%)		0.88 (0.47–1.64)	.683
Medicaid*	13,391 (96.0%)	554 (4.0%)		1.36 (0.72–2.58)	.345
Medicare	83,867 (97.0%)	2553 (3.0%)		1.11 (0.61–2.02)	.732
Charlson/Deyo score					<.001
0	78,945 (97.6%)	1955 (2.4%)	<.001	1	–
1	41,842 (96.8%)	1401 (3.2%)		1.35 (1.26–1.45)	<.001
2	15,985 (97.3%)	613 (2.7%)		1.55 (1.41–1.70)	<.001
3	7507 (95.7%)	336 (4.3%)		1.81 (1.61–2.03)	<.001
Facility setting					.002
Metro	112,152 (97.3%)	3076 (2.7%)	<.001	1	–
Urban	25,072 (96.2%)	990 (3.8%)		1.48 (1.17–1.87)	<.001
Rural	3609 (95.7%)	162 (4.3%)		1.58 (0.93–2.67)	.090
Facility type					<.001
C-CP	18,871 (97.2%)	545 (2.8%)	<.001	1	–
CoCCP	67,169 (97.0%)	2071 (3.0%)		1.33 (0.95–1.86)	.099
ACP	37,298 (96.9%)	1200 (3.1%)		1.81 (1.28–2.56)	<.001
INCP	20,520 (97.7%)	473 (2.3%)		0.58 (0.35–0.95)	.030
Surgery at primary site					
No	143,105 (97.1%)	4279 (2.9%)	.339	–	–
Yes	978 (97.6%)	24 (2.4%)		–	–
Surgery at secondary site					
No	140,722 (97.1%)	4195 (2.9%)	.703	–	–
Yes	3456 (97.0%)	107 (3.0%)		–	–
Radiation					
No	83,608 (97.0%)	2575 (3.0%)	.060	–	–
Yes	56324 (97.2%)	1633 (2.8%)		–	–
Chemotherapy					
No	40,244 (95.5%)	1875 (4.5%)	<.001	1	–
Yes	101,677 (97.7%)	2394 (2.3%)		0.59 (0.48–0.72)	<.001
Immunotherapy					
No	143,144 (97.1%)	4285 (2.9%)	.046	1	–
Yes	712 (98.3%)	12 (1.7%)		0.73 (0.32–1.67)	.456
Metastasis site					
Bone only	1489 (95.8%)	66 (4.2%)	.203	–	–
Brain only	1831 (96.2%)	72 (3.8%)		–	–
Liver only	2768 (96.8%)	92 (3.2%)		–	–
Number of metastatic sites					<.001
1 site only	6088 (96.4%)	230 (3.6%)	<.001	1	–
2 sites	2886 (94.8%)	159 (5.2%)		1.48 (1.19–1.83)	<.001
3 sites	541 (91.9%)	48 (8.1%)		2.47 (1.76–3.45)	<.001

ACP = Academic/Research Cancer Program, CCP = Community Cancer Program, CoCCP = Comprehensive Community Cancer Program, CI = confidence interval, INCP = Integrated Network Cancer Program, NA = not available, OR = odds ratio.

### 3.2. Determinants of referral to PM in NSCLC

Older age was associated with decreased referral to pain specialists, with patients >70 receiving the least amounts of pain referral (OR: 0.77, 95% CI: 0.71–0.83, *P* < .001), followed by patients aged 50 to 70 (OR: 0.90, 95% CI: 0.84–0.96, *P* = .002), as compared to patients <50 years old. Black patients were less likely to receive PM referral as compared to White patients (OR: 0.73, 95% CI: 0.69–0.77, *P* < .001). Interestingly, patients of a race other than White or Black had no differences in PM referral as compared to White patients. Patients who had private insurance received less referrals to PM as compared to uninsured patients (OR: 0.81, 95% CI 0.75–0.88, *P* < .001), while patients on Medicaid or other types of governmental coverage were the most likely to receive referrals to PM. Patients on Medicare had no differences when compared to uninsured patients (OR: 0.94, 95% CI: 0.87–1.02, *P* = .135).

Patients with a higher number of comorbidities as demonstrated by a higher Charlson/Deyo score were increasingly more likely to be referred to pain specialists as compared to patients with no commodities: 1 comorbidity (OR: 1.32, 95% CI: 1.27–1.36, *P* < .001), 2 comorbidities (OR: 1.38, 95% CI: 1.32–1.46, *P* < .001), 3 or more comorbidities (OR: 1.56, 95% CI: 1.46–1.67, *P* < .001). Patients treated in urban (OR: 1.37, 95% CI: 1.31–1.42, *P* < .001) or rural facilities (OR: 1.45, 95% CI: 1.31–1.59, *P* < .001) were more likely to receive PM referrals than those treated in metropolitan facilities. Facility type also played an important role in the likelihood of referrals: patients treated in academic cancer programs were the most likely to be referred (OR: 1.24, 95% CI: 1.17–1.31, *P* < .001), while patients treated at integrated network cancer programs were the least likely (OR: 0.88, 95% CI: 0.82–0.94, *P* < .001).

Moreover, patients who underwent surgical procedures at the primary site were less likely to receive referrals (OR: 0.45, 95% CI: 0.40–0.52, *P* < .001) as compared to those who didn’t receive surgery. Similarly, patients who received chemotherapy were less likely to receive PM referrals compared to those who did not receive chemotherapy (OR: 0.60, 95% CI: 0.58–0.62, *P* < .001). Conversely, patients who underwent radiation were more likely to receive referrals than those who did not receive radiation treatment (OR: 1.32. 95% CI: 1.28–1.37, *P* < .001).

Finally, distant site characteristics played a significant role in the likelihood of referral to PM. Patients with NSCLC metastases to the bone were the most likely to be referred to pain specialists (<0.01) while patients with metastases to the brain were the least likely (OR: 0.49, 95% CI: 0.42–0.58, *P* < .001). Patients with metastases to >1 site were more likely to receive referrals than those who had metastases to only 1 site (*P* < .001). Interestingly, rates were similar when patients had metastases to 2 sites (OR: 1.55, 95% CI: 1.38–1.73, *P* < .001) or 3 sites (OR: 1.55, 95% CI: 1.26–1.92, *P* < .001). Patient sex, ethnicity, surgery at metastatic sites, and immunotherapy did not affect the likelihood of referral to pain specialists (Table 1).

### 3.3. Determinants of referral to PM in SCLC

Age, sex, race, ethnicity, and insurance type were not associated with referral to PM in SCLC. Patients with a higher number of comorbidities, as per the Charlson/Deyo score, were more likely to receive referrals when compared to patients with 0 comorbidity: 1 comorbidity (OR: 1.35, 95% CI: 1.26–1.45, *P* < .001), 2 comorbidities (OR: 1.55, 95% CI: 1.41–1.70, *P* < .001), 3 or more comorbidities (OR: 1.81, 95% CI: 1.81 1.61–2.03, *P* < .001). Facility setting and type also influenced the likelihood of referrals. Facilities located in an urban setting were more likely to issue referrals than metropolitan-located facilities (OR: 1.48, 95% CI: 1.17–1.87, *P* < .001). Conversely, rural facilities showed no difference as compared to metropolitan facilities (OR: 1.58, 95% CI: 9.32–2.67, 0 = 0.090).

Among treatment modalities, only chemotherapy played a role in whether patients received or not a referral to PM. Patients who received chemotherapy were less likely to receive a referral than those who didn’t (OR: 0.59, 95% CI: 0.48–0.72, *P* < .001). The other treatment modalities surveyed – surgical resection of primary sites, surgical resection of secondary sites, radiation, and immunotherapy – did not affect the likelihood of receiving PM referral.

An increase in the number of metastatic sites lead to a significant increase in pain referral: patients with metastases to 3 sites were the most likely to receive a referral (OR: 2.47, 95% CI: 1.76–3.45, *P* < .001) followed by patients who had metastases to 2 sites (OR: 1.48, 95% CI: 1.19–1.83, *P* < .001) as compared to patients with 1 metastatic site only. Interestingly, metastatic site was not a predictor of referral in SCLC (Table 2).

## 4. Discussion

This analysis of patients from the NCDB has identified that 3.1% patients with Stage IV NSCLC and 2.9% of patients with Stage IV SCLC were referred by their treating provider to pain specialists. The importance of these findings becomes evident when data shows that 10 to 20% of patients with pain do not respond to opioid-based therapy or suffer from life-impairing side effects.^[18]^ The latter include constipation, nausea and vomiting, pruritus, delirium, respiratory depression, motor and cognitive impairment, and sedation, which are quite frequent.^[19]^ Moreover, in the era of the opioid crisis,^[21]^ non-opioid PM is an essential consideration; it should nonetheless not come at the price of optimal pain control in cancer patients. The overall low rate of referral in the analyzed patient population puts into question optimal analgesia provision to patients, looking at both the level of pain control and tolerance of the regimen.

As revealed in our study, a higher number of comorbidities – as reflected by the Charlson/Deyo score – lead to higher rates of referral to pain specialists by the treating team in both patient groups. This observation may be driven in part by the higher concern for adverse drug events, particularly drug–drug interaction when prescribing pain medications to this patient population. This concern is well documented in the literature.^[22]^

The National Comprehensive Cancer Network guidelines on Adult Cancer Pain extensively discuss the various methods of PM using both opioid and non-opioid pharmacological therapies. They offer a ladder-based approach to assess pain and escalate treatment using mainly medication, which remains the mainstay of treatment adopted for LC pain management.^[23]^ Conversely, little information and recommendations are available on the indications for specialty consultations for improved PM and interventional strategies for pain relief.^[24]^ Historically considered last resort methods, as reflected by the World Health Organization 3-step Analgesic Ladder,^[25]^ increasing evidence is available on the efficacy of interventional analgesia for pain relief and improved quality of life.^[26]^

Percutaneous catheters that chronically infuse spinal analgesics relieve pain in any targeted part of the body, as determined by their placement.^[27]^ Alternatively, neuroaxial blocks relieve pain associated with nerve compression. Intercostal nerve blocks are also very effective in patients with lung malignancies and mesothelioma.^[29]^ Rhizotomy for chest wall pain from tumor invasion, anterolateral cordotomies, and intrapleural analgesia are also available alternatives to traditional pharmacological analgesia.^[29–32]^ A wide range of interventional analgesia options are available, with different outcomes in different cancer types.

There are other forms of non-pharmacological approaches to PM in patients with LC. These non-pharmacologic approaches include physical interventions, cognitive-behavioral interventions, psychosocial interventions, as well as a variety of spiritual remedies. Such physical interventions include therapeutic or conditioning exercise, physical or occupational therapy, massage, use of heat/cold packs, acupuncture, and acupressure.^[19]^ Cognitive-behavioral interventions include deep muscle relaxation, mindfulness-based stress reduction, breathing exercises, relaxation, imagery, hypnosis, biofeedback, and music therapy.^[19]^ Psychosocial intervention can be given by means of providing support and providing education to patients and their families, which may help enhance the patients’ sense of control and reduce feelings of helplessness. Patients who may hold cultural beliefs may find spiritual practices such as prayer, rituals, or other spiritual practices to help in relieving and/or coping with pain.^[19]^

Gender, age, and sex differences were found when comparing sociodemographic characteristics in Stage IV NSCLC, consistent with the literature documenting disparities in unequal care for pain.^[16,33,34]^ Interestingly, these differences were not found when looking at SCLC. Considering treatment modality, findings for stage IV NSCLC and SCLC were not consistent. Surgery for stage IV NSCLC and chemotherapy for stage IV SCLC predicted a decreased likelihood of referral to pain specialists, while radiation therapy was a predictor of referral to pain specialists. This observation may be explained by the fact that palliative surgery and chemotherapy exacerbate fatigue and cognitive dysfunction in patients, which can lead to severe pain being overlooked as a priority.^[23,35]^ Disparities in access to PM are highlighted when looking at the differences in referrals at the various facility types. In patients with either SCLC or NSCLC, referrals to pain specialists were more common in academic cancer programs, as compared to community cancer programs.

Ineffective PM in patients with LC remains to be an ongoing clinical concern, as current research has revealed that LC pain continues to be under-treated despite the variety of pharmacological and non-pharmacological options.^[36]^ Practices and knowledge managing cancer pain has been documented to be inadequate amongst medical professionals, with the need for more effective interdisciplinary communication to determine appropriate therapies.^[37,38]^ Communication barriers include lack of systematic screening tools/documentation of pain by clinicians, and patient reluctance to discuss their pain due to fear of it interfering with their cancer therapy.^[39,40]^

When evaluating minority patients with LC, unequal care, lower overall referral to pain specialists, and lack of effective pain control were observed in Stage IV NSCLC, consistently across the various treatment cohorts (surgery, chemotherapy, or radiation therapy) and in those who received treatment at community cancer care centers.^[36]^ With the extensive range of options available, multidisciplinary care involving both oncologists and pain specialists becomes essential to provide patients with optimal pain control, especially when keeping in mind both patient comfort and opioid reduction. LC patients who experience refractory pain to traditional analgesics may benefit from interventional PM techniques or other non-pharmacological approaches in PM.^[34]^ The role of pain specialists within palliative care becomes vital in ensuring and providing adequate cancer pain suppression by providing alternative therapies and improving the overall quality of life.

Important limitations of our analysis relate to the retrospective observational study design. While the NCDB confirms there are gaps in the provision of PM in patients with Stage IV LC, it does not outline the reasons behind the observed shortcomings. Future research exploring provider and patient perceptions towards pain treatment using a qualitative approach might shed light on this important topic with additional information. Another limitation is related to the NCDB documentation that lacks other variables that could have played a significant role in patient decision-making, including pain levels, previous experience with PM, and cultural or religious beliefs. Finally, the NCDB only documents patient information based on the time of diagnosis, and no data regarding follow-up are available. There is subsequently no information on whether or not patients who had initially not received referral to pain specialists received PM later on.

Our study highlights the current gap in multidisciplinary palliative care provision for PM in LC. Education of oncology specialists on the assessment and management of pain should be implemented on a national level, with focused awareness of the crucial role of pain specialists in providing safe and effective pain relief to Stage IV LC patients.

## Author contributions

**Conceptualization:** Marita Yaghi, Najla Beydoun, Kelsey Mowery, Sandra Abadir, Maroun Bou Zerdan, Evan Alley, Diana Saravia.

**Data curation:** Marita Yaghi, Rafael Arteta-Bulos.

**Formal analysis:** Marita Yaghi, Hong Liang.

**Investigation:** Marita Yaghi, Najla Beydoun, Kelsey Mowery, Maroun Bou Zerdan, Iktej Singh Jabbal, Carlos Rivera, Rafael Arteta-Bulos.

**Methodology:** Marita Yaghi, Najla Beydoun, Maroun Bou Zerdan, Hong Liang.

**Project administration:** Marita Yaghi, Evan Alley, Diana Saravia.

**Resources:** Marita Yaghi.

**Software:** Marita Yaghi, Hong Liang.

**Supervision:** Evan Alley, Diana Saravia, Rafael Arteta-Bulos.

**Validation:** Evan Alley, Diana Saravia, Rafael Arteta-Bulos.

**Writing – original draft:** Marita Yaghi, Najla Beydoun, Kelsey Mowery, Sandra Abadir, Maroun Bou Zerdan, Iktej Singh Jabbal, Carlos Rivera.

**Writing – review & editing:** Marita Yaghi, Najla Beydoun, Kelsey Mowery, Sandra Abadir, Maroun Bou Zerdan, Iktej Singh Jabbal, Carlos Rivera, Hong Liang, Evan Alley, Diana Saravia, Rafael Arteta-Bulos.
